# Molecular Networking in Cosmetic Analysis: A Review of Non-Targeted Profiling for Safety Hazards and Bioactive Compounds

**DOI:** 10.3390/molecules30193968

**Published:** 2025-10-02

**Authors:** Li Li, Shuo Li, Ji-Shuang Wang, Di Wu, Guang-Qian Xu, Hai-Yan Wang

**Affiliations:** 1 National Institute for Food and Drug Control, Beijing 100050, China; lili_nicpbp@126.com (L.L.);; 2 National Institute of Metrology, Beijing 100029, China; lish@nim.ac.cn; 3 Traditional Chinese Materia Medica, Shenyang Pharmaceutical University, Shenyang 110016, China

**Keywords:** molecular networking, cosmetic quality and safety, non-targeted analysis, illegally added substances, natural active ingredients, tandem mass spectrometry

## Abstract

Molecular networking (MN) is a novel mass spectrometry data analysis method that has advanced significantly in recent years and has rapidly emerged as a popular technique. By visualizing the connections between structurally similar compounds in mass spectra, MN greatly enhances the efficiency with which harmful substances and bioactive ingredients in cosmetics are screened. In this review, we summarize the principles and main categories of MN technology and systematically synthesize its progress in cosmetic testing applications based on 83 recent studies (2020 to 2025). These applications include screening banned additives, analyzing complex matrix components, and identifying efficacy-related ingredients. We highlight MN’s successful application in detecting prohibited substances, such as synthetic dyes and adulterants, with limits of detection (LOD) as low as 0.1–1 ng/g, even in complex matrices, such as emulsions and colored products. MN-guided isolation has enabled the structural elucidation of over 40 known and novel compounds in the analysis of natural ingredients. We also discuss current challenges, such as limitations in instrument sensitivity, matrix effects, and the lack of cosmetic-specific component databases. Additionally, we outline future prospects for expanding MN’s application scope in cosmetic testing and developing it toward computer-aided intelligence. This review aims to provide valuable references for promoting innovation in cosmetic testing methods and strengthening quality control in the industry.

## 1. Introduction

With the progress of science and technology and the rapid rise in living standards, cosmetics have become essential for people to pursue beauty standards and meet their individual needs. The cosmetics industry has rapidly evolved into a massive global market [1]. Consumer demand has gradually shifted from basic skin care to a greater focus on effective, safe, and environmentally friendly and natural ingredients [1,2]. To meet consumer demand, the iteration of cosmetic products has accelerated, with new raw materials, formulations, and concepts emerging continually [3,4,5]. However, this rapid turnover also entails risks: inconsistent product quality, non-compliant ingredient labeling, and the presence of illegal additives, among other issues [6,7,8,9]. As cosmetics are often in contact with the human body for prolonged periods, harmful substances (e.g., allergens, hormonal substances) may trigger allergies, irritation, and other health risks [7,8,10,11,12,13,14]. Therefore, with the widespread use of cosmetics, social concerns about their quality and safety have become increasingly prominent. Ensuring the safety of listed cosmetics is the most basic requirement. Under normal or foreseeable conditions of use, such products must be harmless to human health.

Ensuring the safety of cosmetics and enforcing regulatory compliance requires the development of efficient and robust analytical methods. Chemical analysis is essential for identifying specific raw materials, detecting banned substances, and distinguishing between genuine and adulterated cosmetics. Mass spectrometry (MS), recognized as a “gold standard” in analytical chemistry due to its high sensitivity, rapid analytical speed, and excellent specificity, has been widely used in cosmetic analysis, encompassing compound detection, ingredient screening, and efficacy assessment [15]. However, the complexity of cosmetic matrices, the wide range of risk substances, and the rapid product iteration—all of which result in dynamically changing detection targets—make it challenging for traditional targeted detection methods (based on known risk lists) to keep pace with new raw materials [16,17,18]. On the other hand, existing cosmetic screening technologies still rely heavily on targeted detection using high-performance liquid chromatography–tandem mass spectrometry (LC-MS/MS) based on known standards [19,20,21,22,23,24,25,26,27]. Despite achieving high throughput, these methods still analyze unknown ingredients inefficiently, preventing the accurate detection of structurally modified illegal additives. Therefore, improving the efficiency of non-targeted identification and developing detection technologies with sensitivity, intelligence, and broad-spectrum screening capabilities have become core requirements to ensure the standardized development of the cosmetic market and protect the health rights of consumers. In recent years, high-resolution mass spectrometry (HRMS) has been widely used in pharmaceutical, food, biological, environmental, and cosmetic testing due to its advantages of high resolution, high sensitivity, and non-targeted screening, especially in the identification of illegal additives and screening of active ingredients, which shows a broad application prospect [28,29,30,31,32,33]. However, HRMS generates a large amount of data with complex information, and the existing databases have limitations in spectrum matching and prediction reliability. Enhancing automated data analysis and deep mining, as well as quickly completing structure speculation and confirmation, is an essential challenge for non-targeted analysis.

Tandem mass spectrometry (MS/MS) is a technique that combines first-stage mass spectrometry (MS^1^) and second-stage mass spectrometry (MS^2^) for the purpose of comprehensive molecular characterization. MS^1^ detects intact precursor ions, reporting their mass-to-charge ratios (*m*/*z*) and intensities. This enables the profiling and quantification of ionizable compounds in a sample. This stage thus provides a global view of molecular presence and abundance. In MS^2^, selected precursor ions undergo fragmentation via collision-induced dissociation (CID), thereby generating product ion spectra that function as structural fingerprints. The MS^2^ spectra reveal fragmentation patterns that are directly linked to the molecular architecture of the compounds under investigation. This has been demonstrated to support structural elucidation and confident identification. The combination of two stages facilitates a sequential workflow, from ion detection and screening (MS^1^) to structural confirmation (MS^2^). This workflow constitutes the basis of contemporary untargeted metabolomics, natural product analysis, and small-molecule characterization.

Driven by algorithmic innovations, molecular networking has emerged as the cornerstone methodology for the scalable analysis of high-throughput, non-targeted mass spectrometry datasets. It constructs a compound relationship network by comparing the similarity of MS^2^ of compounds, explores the compounds using databases to infer the structure of unknown substances, and then transforms the complex mass spectral information into intuitive molecular relationship diagrams with the aid of visualization tools [34,35]. Researchers extensively employ this technique to investigate natural products, metabolomics, foodborne hazards, and environmental contaminants, while recognizing its emerging potential for cosmetic safety screening [36,37,38,39,40,41,42,43,44].

Whilst several reviews have previously cataloged the applications of MN in the domains of natural products and metabolomics [34,37], none of these have systematically addressed its specialized utility in the field of cosmetics. This field is characterized by complex matrices (e.g., emulsions, pigments), stringent safety regulations, and rapidly evolving illicit additives. It is imperative to acknowledge that existing literature has hitherto overlooked the integration of artificial intelligence (AI)-driven spectral prediction with MN to resolve the two core challenges in cosmetic analysis: low-abundance active ingredients and structurally modified illegal drugs.

This study conducted a systematic literature search using a multidimensional combination of keywords, including “molecular networking”, “tandem mass spectrometry,” “cosmetic analysis”, “untargeted analysis”, “illegal additives”, “natural active ingredients”, “safety assessment” and “efficacy evaluation” in two authoritative academic databases: PubMed and Web of Science. A comprehensive literature retrieval framework covering the entire process of cosmetic analysis was established. The search period was set from 2020 to 2025 and initially yielded 127 relevant articles. Based on the screening principle that the research topic must closely focus on cosmetic analysis with a standardized experimental design and sufficient supporting data, a dual-independent review and cross-validation process was implemented, resulting in the exclusion of 44 articles due to irrelevant topics or methodological flaws. Ultimately, 83 core articles were included for review and analysis. The scope of application scenarios has expanded to five categories: raw material identification, prohibited substance screening, efficacy validation, matrix interference elimination, and AI integration. This process ensured comprehensive coverage of the literature, fully encompassing key applications of MN in identifying cosmetic ingredients and screening banned substances. This review synthesizes current progress in MS/MS-based MN for cosmetic analysis, critically evaluates persistent challenges, and delineates future directions for AI-driven integration and multi-omics approaches. The sum of these endeavors establishes a forward-looking technological roadmap, with the objective of advancing quality control and innovation in the cosmetic industry.

## 2. MS/MS-Based MN

### 2.1. Principles of MN

The fundamental principle of MN technology is predicated on the notion of structure–spectrum correlation. This principle posits that molecules that are chemically similar yield analogous fragmentation patterns in tandem mass spectrometry [45]. In this technique, the similarity between MS^2^ data vectors is calculated by means of mathematical methods, including the modified cosine similarity algorithm. A network of relationships is constructed between compounds based on the similarity of the MS^2^ spectra, thereby enabling the clustering of structurally similar compounds. The modified cosine similarity algorithm is a solution to the problem of spectral shifts resulting from functional group modifications. It achieves this by comparing neutral mass differences, thereby enhancing consistency in the similarity calculation. This is in accordance with the core assumption that “similar structures lead to similar spectra” [46]. The enhanced spectral similarity search employs a dual comparison strategy in constructing the molecular network. Direct fragment matching is a process that involves the comparison of multiple fragment ion mass-to-charge ratios (*m*/*z*) between two MS^2^ spectra. The purpose of this process is to determine the identity of the molecules in question. This is due to the fact that it enables the detection of molecules with nearly identical chemical structures. This approach successfully circumvents the limitations of conventional fragment matching methodologies by establishing connections between structurally related compounds, that is, those which share functional groups or modifications despite exhibiting disparate parent ion masses. The aforementioned process has been demonstrated to engender an augmentation in the scope of coverage with regard to metabolite families and biological pathways. Furthermore, it has been shown to facilitate the delivery of critical evidence for the purpose of inferring the structure of unknown substances. In addition to this, it has been demonstrated to enhance annotation capabilities [47]. MN has become a central tool for visualizing and annotating chemical space in non-targeted MS data [48].

### 2.2. Development of MN

Researchers initially developed MN for the detection of structurally modified peptides and proteins. In 2007, Bandeira [49] demonstrated that the systematic spectral comparison of overlapping peptides or modified variants enables the reliable identification of proteins and the discovery of novel modified fragments. The “spectral network” concept that he developed provided the foundation for the subsequent evolution of molecular networks. In 2012, Watrous et al. [50] pioneered the use of cosine similarity to calculate the correlation of MS^2^ spectra. They also advanced the field by visualizing the network of MS^2^ similarity and applying this approach to study the metabolic patterns of microbial communities. In 2013, Yang et al. [51] pioneered the conceptual framework of molecular networking. Consequently, they established the open access Global Natural Product Social Molecular Networking (GNPS), which provides online data analysis services and aims to address the issue of low utilization of MS^2^ data in natural product research. The platform enables users to upload MS data to generate molecular networks and integrates public MS databases. The platform has been shown to enhance efficiency in the analysis of unknown compounds through community-based sharing and collaborative annotation. GNPS pioneered a novel paradigm for off-target studies of natural products via MN technology, thereby establishing itself as a core metabolomics tool. The application of GNPS has now expanded into various fields, including but not limited to medical research, plant science, environmental science, food chemistry, and cosmetic science [52].

Classic molecular networking (CLMN), the earliest and most widely used molecular networking method, clusters MS^2^ spectra by directly comparing fragment ions, calculating spectral similarity, and grouping related spectra [53]. The MS^2^ spectra that were obtained were then processed using the MS-Cluster algorithm. This algorithm was used to generate a consensus spectrum by consolidating duplicate spectra. Subsequently, a modified algorithm is employed to calculate the spectral similarity score by comparing peaks between consensus spectra. The construction of a molecular network is predicated on the foundation of these similarity scores. Each network node is representative of a single MS^2^ spectrum. Nodes exhibiting high similarity are linked to form a molecular cluster, known as a “molecular family”. MS^2^ spectra that do not exhibit similarity exist as individual nodes. The algorithms employed in this analytical method are relatively straightforward, facilitating the preliminary classification and clustering of components in complex mixtures. A notable disadvantage associated with the MS clustering algorithm is its inability to differentiate between positional or stereoisomers. This limitation also precludes the provision of accurate relative quantitative information. The system’s vulnerability to spectral noise is a salient concern, as it can lead to a decline in clustering accuracy, particularly in complex matrices or low-intensity ion responses [54].

In order to address the issue of CLMN, GNPS introduced Feature-Based Molecular Networking (FBMN) in 2020. This new approach incorporates multiple types of feature information, including retention time, isotope pattern, neutral loss, and ion mobility, into the traditional molecular network. In comparison with CLMN, FBMN necessitates the utilization of LC-MS/MS data processing software (e.g., MZmine 3, XCMS v4.6.4, Progenesis QI v3.0, MS-DIAL 4) to integrate MS^2^ spectra with retention time, isotope pattern, ion mobility, and other parameters, resulting in the formation of a characterization table (containing *m*/*z*, retention time, intensity/area, correlation, etc.) before the construction of the MN-representative MS^2^ spectra files [54]. Integration and analysis of these features enables FBMN to enhance its capacity to differentiate isomers and structurally similar compounds, thereby ensuring higher accuracy and reliability in complex matrices. Presently, FBMN stands as a pivotal component within the overarching framework of GNPS. Wu et al. [55] systematically analyzed the transformation pathways of five sulfonamides during wastewater bioprocessing using an FBMN-based non-targeted screening strategy. A total of 45 transformation products were identified, including 14 that were newly discovered. The findings indicated that, in comparison with the conventional non-targeting approach, the FBMN strategy exhibited notable advantages in the prioritization of candidate transformation products and the identification of novel products, particularly in the context of low-abundance transformation products.

LC-MS/MS ionization generates a multitude of ionic species from individual compounds, encompassing protonated molecules ([M+H]^+^) and sodiated adducts ([M+Na]^+^). The molecular network is constructed by ions with distinct mass-to-charge ratios (*m*/*z*), resulting in the observation of discrete nodes. A substantial body of research has demonstrated that the fragmentation behavior of these prevalent ion adducts exhibits considerable variation during collisional activation processes, such as collision-induced dissociation (CID). Additionally, networks derived exclusively from MS^2^ spectra may lack the capacity to correlate all the ion adducts produced by a given compound. This phenomenon frequently results in the unnecessary fragmentation of molecular families (sub-networks), which limits the efficiency of propagation of spectral library annotations through the network. To address this challenge, Schmid et al. developed ion identity molecular networking (IIMN), a method that integrates chromatographic peak shape correlation analysis into molecular networks to connect and merge different ionic species of the same molecule [56]. The novel feature relationships serve to enhance network connectivity among structurally related molecules. These tools facilitate the identification of novel ion–ligand complexes, enhance the accuracy of annotations within molecular networks, and promote the expansion of spectral reference libraries. Despite being integrated into open-source feature annotation tools and GNPS platforms, the IIMN is entirely reliant on the comprehensiveness and accuracy of its database. This reliance imposes limitations on its utility for detecting novel compounds or uncharacterized structural modifications that are not represented in reference libraries.

The bioactive molecular network (BMN) is an alternative technology derived from FBMN, whose core goal is to rapidly localize clusters of compounds with specific bioactivities and guide target discovery and mechanism studies of active ingredients through molecular networks constructed based on similarities in MS^2^ spectra (e.g., fragmentation ion matches, neutral loss modes) in conjunction with the results of bioactivity screening of compounds (e.g., antimicrobial, anti-inflammatory, anticancer, and other activity data) [57]. In 2023, researchers pioneered an integrated FBMN-BMN strategy to predict anthelmintic constituents in Morinda lucida Benth. This novel methodology rapidly pinpointed 16 potential bioactive compounds, including unprecedented cyclic enol ether terpenes and flavonoids. Crucially, the research team identified strychnine acid and kaempferol-3-O-rutinoside as key anthelmintic agents. This integrated approach not only accelerates the qualification process but also reduces research and development costs [58].

To overcome the shortcomings of traditional molecular network techniques in identifying compounds with novel backbone structures, Qi-Fang He’s group recently proposed, for the first time, a building block-based molecular network (BBMN) strategy by integrating the biogenic block identification technique and the molecular network technique [59]. Compared to traditional molecular network technology, the BBMN strategy offers clear advantages in discovering compounds with novel backbone structures: for one, it can quickly identify the biogenic building blocks in complex extracts based on the structural features of the target compounds, providing strong selectivity for the analyzed compounds; furthermore, due to the large volume and redundancy of secondary MS data, BBMN can reduce the analyte dataset through selective filtering and visualize it using MN, making it easier for researchers to rapidly target specific compounds.

A comparative analysis of the five MN technologies reveals that they each have distinct focuses in terms of their core principles (Table 1). For instance, CLMN performs clustering based on the similarity of MS^2^ spectral fragment ions. FBMN integrates multiple features, and IIMN correlates different adduct ions. It is evident that each method possesses a distinct set of advantages and limitations, rendering them suitable for specific, ideal application scenarios. CLMN is applicable to preliminary mixture screening; however, it is incapable of resolving isomers. FBMN is capable of fine-scale analysis of complex systems, such as plant extracts, yet it relies on preprocessing software. BMN facilitates the discovery of bioactive ingredients but requires additional bioassays. BBMN has high selectivity for the discovery of novel scaffolds in fields such as microbial metabolites, though it depends on biosynthetic rule libraries. A thorough examination of these technologies reveals that they exhibit complementary characteristics and specific applicability.

### 2.3. Molecular Network Construction Process

**Figure 1:**
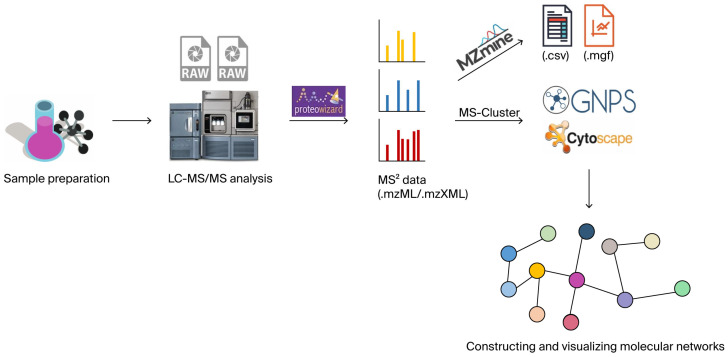
MS/MS-based molecular networking workflow. Colored bar charts represent MS^2^ data, recording the fragment ion information of individual molecular features from mass spectrometric analysis. The bar height corresponds to the relative abundance (or peak intensity) of the corresponding fragment ions, with distinct colors distinguishing between sample sources or detection batches. The spherical component represents the constructed molecular network, where each ball denotes a molecular feature node—ball color indicates different compound categories (e.g., alkaloids, flavonoids), and ball size reflects node connectivity (or matching confidence between the molecular feature and reference database entries).

Molecular network construction involves the following key steps (Figure 1):

(1) Sample preparation, including extraction and purification. Matrix interferences are removed using liquid–liquid extraction (LLE), a sample pretreatment method that is quick, easy, cheap, effective, rugged, and safe (QuEChERS), and solid-phase extraction (SPE), while quality control samples are prepared to ensure the reliability of the data.
(2) MS/MS data acquisition in a data-dependent mode using LC-MS/MS or LC-HRMS with different collision energy gradients to cover compounds of varying stability.
(3) Raw data are converted to mzML/mzXML formats using tools such as ProteoWizard v3.0.23246, then imported into MZmine 3, MS-DIAL 4, or equivalent platforms for chromatographic peak detection and peak list alignment. These operations yield a comprehensive feature table (.csv format) containing mass-to-charge ratios (*m*/*z*), retention times, peak areas, and cross-sample correlation metrics, alongside representative processed MS^2^ spectral files (.mgf format) [60].
(4) The finalized feature table (.csv) and processed spectral files (.mgf) are then co-submitted to GNPS, where the platform autonomously constructs molecular networks based on MS^2^ spectral similarity thresholds.
(5) After constructing the molecular network on GNPS, the graph file is exported (e.g., .graphml) and the analysis results are visualized using Cytoscape v3.10.2 software to refine network topology and annotate nodes/edges.
(6) Using the GNPS data platform for molecular network analysis, structural analogs of known and undiscovered compounds are inferred and identified based on topological relationships between molecular nodes.

## 3. Computational Framework, Integration Challenges, and Future Perspectives of MN

### 3.1. Algorithmic Foundations of MN

As a core computational technique driving MS data interpretation, MN relies on structured algorithmic workflows to transform high-dimensional MS^2^ data into interpretable chemical networks. The MN workflow is predicated on three sequential algorithmic steps, each of which is optimized to extract chemical relationships from noisy MS/MS data [34,35,45,46].

The algorithm’s initial procedure involves the implementation of data purification on raw MS/MS spectra. The process entails the filtration of noise based on a signal-to-noise (S/N) ratio of ≥3:1, the correction of baselines, and the calibration of *m*/*z* values (with a tolerance of ±0.01 Da) using an internal standard (e.g., *m*/*z* 195.0876). Concurrently, it eliminates weak peaks—defined as those with intensities below 5% of the intra-cluster average intensity or absolute counts fewer than 100.

Subsequently, the algorithm calculates inter-spectral similarity using modified cosine similarity, assigning higher weights to high-intensity peaks. The preliminary screening process then identifies potential duplicate spectra based on a predefined threshold (typically ranging from 0.75 to 0.85, reduced to 0.7 for complex matrices). Clustering is executed via the seed spectrum method, contingent on the stipulation that the average intra-cluster similarity must not be less than 0.65. In the event that this criterion is not met, cluster splitting is initiated to ensure reliability. In the process of generating representative spectra, the median intensity is calculated for peaks that are shared by at least 80% of the spectra within a given cluster. Peaks with intensities exceeding 10% of the median value, along with auxiliary characteristic peaks present in 50% to 80% of the spectra, are retained for further analysis. The efficacy of the algorithm is evident in its ability to enhance the signal-to-noise ratio of the output spectra, achieving a minimum enhancement of 2-fold. Furthermore, it ensures the preservation of over 90% of the original characteristic peaks, thereby demonstrating its capacity to preserve spectral integrity. The output of the algorithm encompasses several components, including representative spectra, the sources of the original spectra, and clustering parameters. These components are formatted in standard formats, such as mzML and MGF, to facilitate downstream analysis. The algorithm’s implementation results in a reduction in data redundancy, an enhancement of the signal-to-noise ratio, and an improvement in the structural representativeness of the data.

Subsequent to this, all spectrum pairs with similarity scores surpassing the predefined threshold are linked to establish a network graph. Subsequently, community detection algorithms (e.g., the Louvain method) are employed to cluster the network, thereby dividing highly interconnected node groups into distinct “molecular families.” These families are analogous to structural analogs that possess shared backbones or functional groups. Finally, visualization is conducted using tools such as Cytoscape, which enable researchers to intuitively explore the chemical space and rapidly identify target compound families.

### 3.2. Challenges and Strategies for Integrating Diverse Datasets

As research increasingly relies on data generated across different instruments, laboratories, and experimental conditions, the integration of multi-source mass spectrometry (MS) data into a unified molecular network presents both significant opportunities and substantial challenges. High-resolution MS instruments, including Orbitrap and Fourier transform ion cyclotron resonance (FT-ICR) mass spectrometers, offer mass accuracy at the sub-ppm level. This capability confers substantial advantages in the identification of isobaric compounds and the determination of molecular formulas. Conversely, quadrupole or time-of-flight (TOF) analyzers, despite their cost-effectiveness and rapid scanning speeds, exhibit comparatively diminished mass accuracy. This renders them well-suited for scenarios where rapid scanning is prioritized over extreme precision [47]. Consequently, in studies necessitating high-precision mass data, such as the annotation of unknown metabolites or the de-duplication of natural products, meticulous selection of MS platforms is paramount. Furthermore, variations in liquid chromatography conditions, including column chemistry (e.g., C_18_ vs. HILIC), gradient profiles, flow rates, and temperature, have been demonstrated to cause retention time drift or compression. This complicates peak alignment and feature matching across datasets.

In the context of MN analysis, the integration of multi-source data is contingent upon a systematic and standardized workflow. Calibration is a critical first step. Instrumental drift in mass spectrometry can lead to inaccuracies in *m*/*z* values and retention times over time. To address this, internal standards such as caffeine, lidocaine, or reference lipids should be used to calibrate both parameters. Accurate calibration is paramount for ensuring high mass precision, typically within the range of ±0.01 Da. This process also facilitates the alignment of retention times across runs, a crucial step in the analysis process. This alignment facilitates the execution of meaningful comparisons between datasets and assists in the prevention of misinterpretations that may arise from instrument-specific variations.

Maintaining uniformity in the identification of features is of equal importance. In order to ensure the integrity of the research, it is imperative that researchers employ a uniform software platform (e.g., MZmine 3, XCMS v4.6.4, or OpenMS v3.0.0) and implement identical settings for critical processing steps, including peak picking, deisotoping, adduct grouping, and cross-sample alignment. This uniform approach is essential for ensuring the consistent detection and representation of molecular features, such as the *m*/*z* and fragment ions, across all samples. In the absence of consistency, the identification of a given compound may vary depending on the dataset, potentially compromising the integrity of the analysis.

Spectral matching constitutes a fundamental element of MN analysis, and the modified cosine similarity score is endorsed as a means to achieve this objective. This method has been extensively implemented in platforms such as GNPS, and it has been shown to enhance the accuracy of basic cosine similarity by accounting for neutral losses and common adducts, such as [M+Na]^+^ or [M+H]^+^. Consequently, it facilitates more robust comparisons between spectra, even when they are acquired under disparate experimental conditions. In the context of heterogeneous datasets, generated on disparate instruments or in varied laboratories, a modest reduction in the similarity threshold, from the conventional 0.8 to a range of 0.6 to 0.7, has been demonstrated to enhance sensitivity. A lower threshold has been demonstrated to enhance the probability of detecting structurally related compounds and identifying potential matches that might otherwise be overlooked. However, it is imperative to exercise caution when implementing this approach. In order to circumvent the potential for erroneous linkages, wherein unrelated spectra are erroneously connected, it is imperative to undertake manual verification of pivotal matches. This precautionary measure is instrumental in ensuring the integrity and precision of the network.

### 3.3. Integration Prospects with Deep Learning Methods

Despite its strengths, MN faces significant challenges in annotating unknown compounds, resolving components within complex mixtures, and distinguishing structurally similar isomers or homologs. These tasks often require expert knowledge and high-quality reference spectra. These limitations underscore the necessity for more intelligent and automated approaches. Typically, MN relies on cosine similarity to establish connections between nodes based on MS/MS spectral matches. However, this approach is sensitive to spectral quality. Low-intensity signals, high levels of noise, or incomplete fragmentation patterns can lead to failed matches or false linkages. These limitations impede the comprehensiveness and precision of the resulting network.

To address these limitations, deep-learning-based algorithms have been developed, including Spec2Vec [61] and MS2DeepScore [62]. Deep learning, a sophisticated branch of machine learning, draws inspiration from the structure and function of the human brain by constructing multi-layered artificial neural networks—so-called “deep models” —that can automatically learn complex features and patterns from large volumes of data. In the realm of scientific research, particularly in the fields of chemistry and biology, deep learning has witnessed a marked surge in popularity for a range of applications, including spectral interpretation, molecular property prediction, and drug discovery. Deep learning provides a robust solution by enhancing both spectral quality and similarity assessment. Firstly, deep models can be applied to preprocess raw spectra, thereby effectively denoising signals and imputing missing fragment ions. This, in turn, improves the overall quality of low-confidence spectra. Moreover, in contrast to the utilization of handcrafted similarity metrics, deep learning has the capacity to directly learn a latent representation of chemical similarity from large spectral datasets. Consequently, deep learning-enhanced matching has been shown to enhance the detection of true spectral similarities, particularly for low-abundance or poorly fragmented compounds, resulting in more comprehensive and accurate molecular networks.

Sheng et al. [63] compared four advanced spectral similarity algorithms. These included modified cosine similarity with shifted peak matching, MS2DeepScore, Spec2Vec, and entropy similarity. The objective of the study was to construct molecular networks for the identification of compounds that are not represented in reference libraries. The Spec2Vec algorithm demonstrated the most optimal performance in detecting structurally analogous analogues and exhibited a reduced false discovery rate. Subsequent evaluation confirmed that Spec2Vec-based molecular networking is a robust and efficient method for rapidly screening illegal adulterants in dietary supplements and herbal medicines.

A significant constraint of conventional molecular networking is its reliance on spectral database matching for compound annotation. By training deep models on extensive, annotated MS/MS datasets, these systems can learn to associate spectral fragmentation patterns with particular molecular substructures, functional groups, or even core scaffolds. Remarkably, such models have the capacity to predict structural features directly from fragment ion data, obviating the necessity of a direct match to a reference spectrum. This capability enables the inference of chemically meaningful attributes, such as the presence of hydroxyl, glycosyl, or aromatic moieties, even for entirely novel compounds. Consequently, it effectively expands the coverage of current spectral databases [64]. It is important to note that, once trained, deep learning models are capable of processing vast amounts of MS/MS data in seconds. This has the effect of dramatically accelerating annotation workflows and rendering large-scale, real-time metabolite profiling increasingly feasible.

## 4. Applications of MN in Cosmetic Raw Material Exploration and Risk Substance Detection

### 4.1. Analysis of Naturally Active Ingredients

Natural active ingredients have become the primary focus for developing modern cosmetic raw materials due to their excellent biocompatibility, low irritation, and multifunctionality [65]. Consumers’ increasing concerns around product safety and environmental sustainability have led to studies on natural ingredients derived from plant extracts, microbial fermentation products, and biologically active marine substances, which have demonstrated significant advantages in functional areas such as skin lightening, anti-aging, and soothing [66,67,68]. However, the complexity of natural extracts poses considerable challenges for identifying and characterizing all active ingredients.

MN constitutes a powerful tool for characterizing naturally active molecules in cosmetics (Table 2). It not only aids in identifying known active ingredients but also enables the recognition of new or uncharacterized molecules in natural extracts. Within networks constructed from MS/MS data of cosmetics containing natural extracts, nodes representing active molecules can be clustered into molecular families. Comparative analysis of node spectra against public or in-house spectral libraries enables rapid construction of compound association networks for complex natural materials. The visualization of intermolecular association maps accelerates the identification of synergistic bioactive clusters and their interaction mechanisms—significantly shortening the discovery cycle for novel compounds. For example, MN of a plant extract-based toner revealed an antioxidant molecular family containing both characterized flavonoids and uncharacterized structural analogs, suggesting new active ingredients with potential antioxidant properties [69].

Hughes et al. [70] investigated three haircare plants from French Polynesia: the aerial parts of *Bidens pilosa*, the leaves of *Calophyllum inophyllum*, and the fruits of *Fagraea berteroana*. Recognizing the critical role of dermal papilla cell proliferation in hair follicle morphogenesis, the team implemented BMN correlating LC-MS/MS features with proliferation metrics through Pearson correlation coefficients. This approach identified key hair-growth stimulants: glycosylated flavonols and phenolic acids in *B*. *pilosa* and *C*. *inophyllum*, along with specialized C-flavonoids, iridoids, and secoiridoids in *F*. *berteroana*. These findings enable the molecular elucidation of traditional botanical mechanisms that promote follicular development, thereby establishing a scientific foundation for innovations in natural hair care.

In response to skin damage and pathological conditions caused by oxidative stress, Chambon et al. [71] systematically evaluated the antioxidant activity of five traditional Polynesian medicinal plants (*Calophyllum inophyllum*, *Gardenia taitensis*, *Curcuma longa*, *Cordia subcordata*, and *Ficus prolixa*). LC-MS/MS metabolomics identified 61 metabolites. GNPS-based molecular networking (positive-ion mode, singleton nodes removed) revealed novel compound clusters, among which some compounds from *Cordia subcordata* and *Ficus prolixa* were reported for the first time. Multi-index evaluations—total phenolic content, 2,2-Diphenyl-1-picrylhydrazyl (DPPH) free radical scavenging, and Ferric reducing antioxidant power assay (FRAP) reducing capacity—showed that the extract from the aerial roots of *Ficus prolixa* exhibited the most vigorous activity, and cellular-based antioxidant power 1 (AOP1) assay confirmed its intracellular antioxidant effect. Online HPLC-DPPH coupling technology further identified seven phenolic substances, including quercetin-*O*-rhamnoside, rosmarinic acid, and curcumin, as key components in the free radical scavenging process. This study was the first to reveal the significant application potential of *Ficus prolixa* as a novel type of skin antioxidant.

Kim et al. [72] applied a BMN strategy to discover bioactive components in *Celastrus orbiculatus Thunb* fruits, which exhibit significant inhibitory effects on α-melanocyte-stimulating hormone (MSH)-induced melanin production in B16F0 melanoma cells. Within the molecular network, nodes exhibiting elevated bioactivity scores received priority for isolation. As a result, 12 unreported dihydro-*β*-agarofuran sesquiterpenes and 15 known compounds were isolated from the methanol (MeOH) extract of *Celastrus orbiculatus* fruits. Their structures were unambiguously determined by analyzing nuclear magnetic resonance (NMR), high-resolution electrospray ionization mass spectrometry (HRESIMS), electronic circular dichroism (ECD), and single-crystal X-ray diffraction data. The team also isolated 7 previously undescribed dihydro-*β*-agarofurans and 28 known compounds from *Celastrus orbiculatus* fruits using LC-MS/MS-based molecular network-guided purification. Their structures were elucidated via 1D and 2D nuclear magnetic resonance, HRESIMS analysis, and ECD quantum chemical calculations. All isolated compounds were tested for their inhibitory effects on α-MSH-induced melanogenesis in B16F0 melanoma cells [73].

Marine red algae, which synthesize unique secondary metabolites to resist UV radiation, serve as high-quality sources of natural sunscreens and antioxidants, holding significant value in cosmeceutical applications. Zwerger et al. [74] established a modern metabolomic analysis strategy, employing UHPLC-HRMS combined with FBMN to systematically analyze algal natural products, focusing on mycosporine-like amino acids (MAAs). The study integrated multi-dimensional databases through the GNPS workflow and optimized the identification process with chemotaxonomy and fragment behavior analysis. It achieved high-throughput visualization and de-duplication detection of active components in red algae for the first time, providing new methodological support for developing red algae-derived photoprotective agents.

Microalgae, as sustainable sources of high-value bioactive peptides, show significant potential in cosmetic applications. Studies by Masoumifeshani et al. [75] have demonstrated that *Arthrospira platensis* and *Chlorella vulgaris* can yield 82% and 72% crude protein, respectively, through alkaline extraction. The <3 kDa peptide fragments produced after enzymatic hydrolysis exhibit excellent bioactivity, with antioxidant and anti-aging effects. Based on mass spectrometry and GNPS molecular networking analysis, eight dipeptides/tripeptides, such as Lys-Val, Val-Arg, Tyr-Phe, and Leu-Gly-Leu, were identified as key active components. This finding confirms that microalgal enzymatic hydrolysate peptides can serve as innovative raw materials for antioxidant and anti-aging cosmetics, providing a new pathway for the development of environmentally friendly cosmetics.

The deterioration of cellulite (more common in women) is associated with factors such as stress, obesity, and aging, highlighting an urgent need for the development of effective intervention strategies. Hegazi et al. [76] systematically analyzed the metabolomic diversity of fruits from nine Apiaceae plants and screened their antioxidant and anti-cellulite activities using UPLC-HRMS/MS combined with MN. The results showed that extracts from *Apium graveolens* and *Petroselinum crispum* significantly increased lipolysis, inhibited adipogenesis, and exhibited excellent free radical scavenging capacity. Molecular network identification revealed that apigenin and its glycoside components are the primary active substances, which may synergistically mediate antioxidant effects and regulate lipid metabolism. This finding confirms that extracts of these two Apiaceae plants have the potential to be developed into anti-cellulite cosmetics, providing new candidates for naturally derived cellulite management agents.

Buche et al. [77] systematically evaluated the biological activities of aqueous extracts from three French oak species. The polyphenol compositions were analyzed using UHPLC-HRMS combined with MN, and their antioxidant and anti-enzyme activities were correlated. The results showed that all three extracts significantly inhibited collagenase activity, and *Q. pubescens* was confirmed to have equivalent efficacy for the first time. Semi-preparative HPLC fraction analysis revealed that the total antioxidant activity originated from the synergistic effect of polyphenolic components, among which condensed tannins and flavonol glycosides were the key functional molecular groups. MN clarified that substances such as quercetin derivatives, ellagic acid, and procyanidin B_2_ delay skin aging through a dual mechanism of scavenging reactive oxygen species (ROS) and inhibiting proteases. This study provides a scientific basis for the ingredient action mechanism in developing anti-aging cosmetics using oak extracts.

Son et al. [78] investigated the secondary metabolites of *Inula japonica* leaves and evaluated their potential in combating skin aging. Leveraging LC-MS-based molecular networking and compound isolation techniques, the study pinpointed caffeoylglucaric and caffeoylquinic acids as the main constituents of the 30% ethanolic extract from *I. japonica* leaves (IJE). Notably, the chemical structures of three previously undescribed caffeoylglucaric acids, named inulajaponic acids A–C, were successfully elucidated. Their subsequent research demonstrated that the newly discovered compound can regulate inflammatory cytokines, prevent collagen degradation, and effectively alleviate inflammatory responses associated with skin aging.

### 4.2. Identification of Prohibited Ingredients and Risk Substances

MN technology has been widely applied in screening risk substances in the environmental, medicine, and food safety fields. For example, non-targeted clustering analysis identifies pharmaceuticals, such as antibiotics and their transformation products [79], as well as emerging environmental pollutants [80]. It enables the simultaneous identification of illegal pharmaceutical ingredients [39], structurally related impurities, and adulterants, thereby improving the efficiency of substance identification. In food risk monitoring, non-targeted methods based on MN are used to screen for known and unknown natural toxins in various foods [81]. Combined with deep learning-based similarity algorithms, MN is employed to rapidly screen and identify illegal adulterants in dietary supplements and herbal medicines [63].

In cosmetic risk substance screening, MN exhibits particularly significant technical advantages. Prohibited colorants in cosmetics are subject to strict regulatory control due to potential health risks (such as carcinogenicity and sensitization). However, traditional targeted detection methods (e.g., HPLC) have obvious limitations: their detection range is restricted by the structures of known target substances, making it challenging to cover structurally unknown or new types of illegal additives, easily leading to false-negative results due to missed detection. To address this issue, Woo et al. [82] were the first to couple MN technology with liquid chromatography–quadrupole time-of-flight high-resolution mass spectrometry (LC-Q-TOF-MS). They developed a solution for addressing the challenge of non-targeted colorant screening (especially structurally similar ones) in complex cosmetic matrices. This coupled technology shows outstanding performance in non-targeted colorant identification: in the detection of eyebrow tattoo products, it successfully detected three structurally similar prohibited colorants (with accurate mass-to-charge ratios of *m*/*z* 267.116, 315.149, and 345.157, respectively). Through cluster analysis of the molecular network, these substances were confirmed to belong to the same family of azo dye derivatives. Compared with traditional methods, this technology has significant advantages in avoiding false negatives: traditional targeted detection often misses unknown colorants if corresponding standard substances are lacking; in contrast, MN technology can associate unknown substances with known compound families through the similarity of fragment ions, enabling the identification of potential risk substances without relying on standard substances, thus significantly improving screening coverage. In summary, the combination of MN and LC-MS/MS provides a reliable means for the rapid characterization and efficient screening of non-targeted colorants in cosmetics, and its technical advantages have been verified in practice.

**Figure 2:**
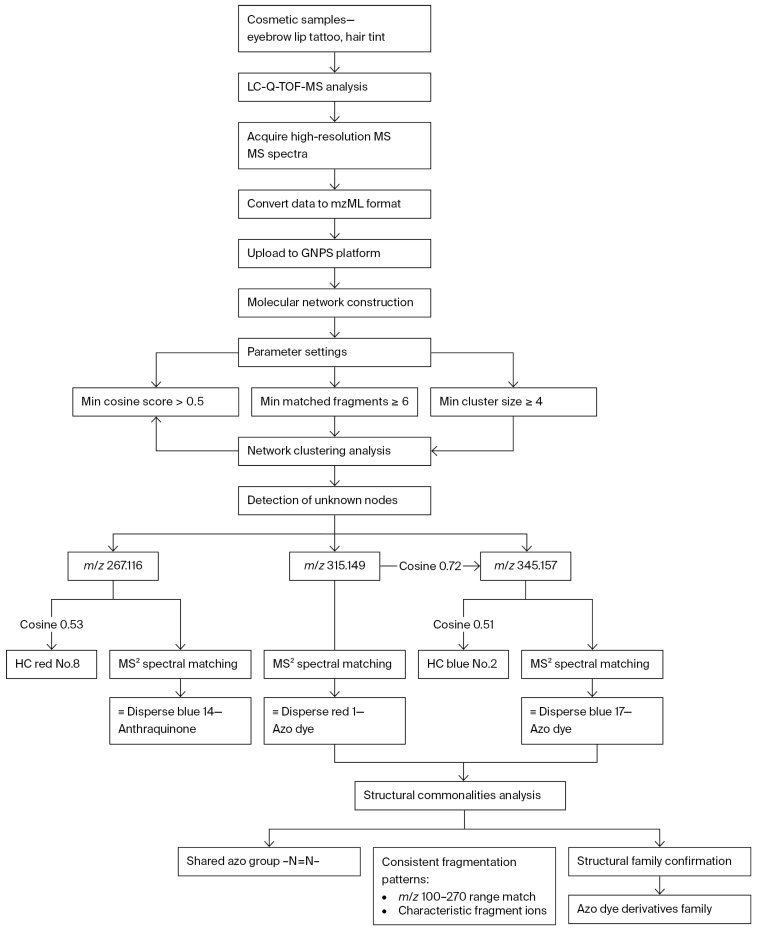
Workflow for non-targeted screening and structural confirmation of banned azo dye derivatives in cosmetics using LC-Q-TOF-MS with MN. This workflow comprises four key stages: (1) Acquisition of high-resolution MS/MS spectra for cosmetic samples via LC-Q-TOF-MS. (2) Construction of MNs. (3) Discovery of unknown compounds through MNs. (4) Structural confirmation of unknown compounds via MS^2^ spectrum matching.

The process consists of four key phases (Figure 2): (1) Sample Analysis: Cosmetic samples (eyebrow tattoo, lipstick tattoo, hair tint) undergo LC-Q-TOF-MS to acquire high-resolution MS/MS spectra. (2) MN: Converted mzML data are processed in GNPS with critical parameters (min. cosine score > 0.5, min. matched fragments ≥ 6, min. cluster size ≥ 4) to construct similarity networks. (3) Unknown Detection: Three unidentified nodes (*m*/*z* 267.116, 315.149, 345.157) show high connectivity (cosine 0.51 to 0.72) to known colorants. (4) Structural Confirmation: MS^2^ spectral matching identifies them as Disperse Blue 14 (anthraquinone), Disperse Red 1, and Disperse Red 17 (azo dyes). Shared fragmentation patterns (*m*/*z* 100 to 270 range) and azo functional groups (-N=N-) confirm their classification as azo dye derivatives. This approach enables reference-standard-free identification of structurally related banned colorants.

## 5. Challenges and Optimization Strategies

### 5.1. Challenges in MS Data Quality

MN platforms, such as GNPS, depend on high-quality MS^2^ data but face significant challenges in trace-level analysis of cosmetics. Target analytes often exist at trace concentrations, resulting in low signal-to-noise ratios (S/N) in MS^2^ spectra. This compromises the processes of spectral feature extraction and molecular network integration, increasing the risk of missed detections or misidentifications. Poor separation of analytes and suboptimal peak shapes also compromise data reliability.

CLMN relies solely on MS^2^ scanning and can only provide qualitative results. Despite the fact that FBMN, an integration of primary MS and chromatographic data, achieves approximate quantification, it is unable to meet the requirements of strictly calibrated quantification [54]. Consequently, when a study necessitates precise quantitative data, subsequent verification through LC-MS/MS remains imperative.

Furthermore, when the number of product ions generated by the cleavage of precursor ions is minimal (e.g., less than four), the complexity of network construction and spectral library matching increases considerably. According to the principles of spectral false discovery rate estimation, the interpretation of low-abundance fragment ion spectra necessitates a heightened degree of caution to mitigate the potential for misjudgment resulting from an inadequate number of features [47].

### 5.2. Methodological Limitations of MN for Structurally Modified Adulterants

Current MN workflows demonstrate proficiency in the structural elucidation of natural products; however, they are deficient in addressing the detection of structurally modified illegal adulterants (e.g., sulfonamide analogs) in cosmetics. These findings are predicated on two fundamental limitations: The conventional similarity algorithms demonstrate a lack of sensitivity to minor chemical moiety differences. Furthermore, no MN framework has been validated for complex cosmetic matrices. This discrepancy hinders the application of MN in real-world cosmetic safety screening, where structural analogs of banned substances are becoming increasingly prevalent.

### 5.3. Matrix Interference in Cosmetic Analysis

Cosmetics are composed of intricate matrices that, in addition to active ingredients and potential risk substances, contain various components such as emulsifiers, thickeners, preservatives, and solvents. These matrix components have the potential to interfere with MS/MS analysis and the subsequent construction of MN. To overcome the challenges posed by matrix interference, a range of sample preparation methods are employed. Prior to MS/MS analysis, conventional methods such as liquid–liquid extraction, solid-phase extraction, and gel permeation chromatography are frequently employed to separate target compounds from the matrix components [83]. However, these pretreatment methods may not eliminate matrix interference and can be time-consuming and labor-intensive. The development of more effective strategies to address complex matrix interference in cosmetic analysis remains a significant challenge.

### 5.4. Limitations of Spectral Databases in Cosmetic MN

The performance of MN in cosmetic analysis is contingent upon the quality and comprehensiveness of spectral databases. These databases are utilized for the purposes of comparison and annotation. Presently, public spectral databases function as foundational resources for chemical analysis, particularly in the context of mass spectrometry. Prominent examples include the National Institute of Standards and Technology (NIST) Mass Spectral Library, GNPS, MassBank, MetLin, and PubChem. These databases play a critical role in supporting the physicochemical analysis of cosmetic products. The National Institute of Standards and Technology (NIST) library is a valuable tool for the matching of gas chromatography-mass spectrometry (GC-MS) data. It has the capacity to generate a list of potential compounds, thereby facilitating the identification and analysis of chemical substances. Furthermore, the retention index data from the NIST library can be queried to provide additional validation of the GC-MS results. In the context of HPLC-MS data from cosmetic samples, two predominant approaches are frequently employed. Initially, MS/MS spectra can be matched using MassBank or MetLin. Secondly, potential molecular formulas can be calculated based on exact mass values. Subsequent to the identification of initial compounds, their nomenclature or Chemical Abstracts Service (CAS) numbers are to be entered into PubChem. Comprehensive information, including physicochemical properties, toxicological data, and regulatory status, is then accessed through this database.

Each of these databases has a distinct focus, resulting in limitations in terms of coverage. This assertion is particularly salient in the context of the diverse array of substances found in cosmetics. Cosmetics contain a variety of distinctive compounds. The following substances are included:

- Polymeric substances resulting from recent synthesis, utilized in formulations;
- Specific natural products derived from rare plant species Existing databases frequently contain an insufficient amount of data regarding these compounds.

The aforementioned factors complicate the accurate identification of the subjects in question by means of MN. To illustrate this point, consider the following example: a novel natural extract is incorporated into a cosmetic product, yet its active molecules are not recorded in any database. In such instances, MN’s capacity to furnish reliable identification results for these molecules may be compromised. In order to enhance the precision and relevance of MN, it is imperative to expand the spectrum of existing databases to encompass a more extensive array of compounds related to cosmetics.

Each of these databases has a distinct focus, resulting in limitations in terms of coverage. This assertion is particularly salient in the context of the diverse array of substances found in cosmetics. Cosmetics contain a variety of distinctive compounds. These include newly synthesized polymers used in formulations and specific natural products from rare plants. Existing databases frequently contain an insufficient amount of data regarding these compounds. This complicates the identification process using MN. For example: a novel natural extract is incorporated into a cosmetic product, yet its active molecules are not recorded in any database. In such instances, the reliability of MN’s capacity to furnish identification results for these molecules may be compromised. To enhance the precision and relevance of MN, it is imperative to expand the spectral databases to encompass a greater number of cosmetic-related compounds.

### 5.5. Optimization Strategies for MN in Cosmetic Analysis

In order to mitigate the effects of interference from complex cosmetic matrices, advanced sample preparation techniques are essential. Online solid-phase extraction (Online SPE) facilitates the integration of extraction, purification, and enrichment processes, thereby effectively suppressing the effects of emulsifiers and polymers in the matrix. Dispersive solid-phase extraction (d-SPE) employing advanced sorbents—such as zirconia-modified magnetic particles or graphitized carbon black—selectively removes interferences, including dyes, oils, and fragrances, significantly improving the recovery of target analytes. These approaches have been shown to enhance sample purity while optimizing chromatographic resolution and mass spectrometric reproducibility [17].

Beyond sample preparation, hybrid mass spectrometry strategies improve spectral quality. For example, combining data-dependent acquisition (DDA) with high-resolution accurate mass (HRAM) scanning enables the capture of more fragment ions, even for trace-level compounds. This approach is crucial for addressing the “low-fragment-ion challenge” (i.e., fewer than four product ions). Conventional cosine similarity metrics often fail to distinguish true matches from random spectral matches in such cases.

Finally, it is imperative to advance algorithmic innovations to better adapt molecular networking (MN) to the unique challenges posed by cosmetic analysis. Specifically, a dedicated “cosmetic matrix interference spectral library” can be embedded into existing deep learning models, such as Spec2Vec and MS2DeepScore, to automatically recognize and suppress characteristic fragments originating from common matrix components (e.g., emulsifiers, silicone oils). This would significantly reduce false-positive identifications in highly interfering samples [47]. Furthermore, to address the difficulty in annotating spectra with few fragment ions, a structure-guided spectral enhancement algorithm should be developed. Such an algorithm would infer the core structure of unknown compounds based on limited fragmentation data and perform integrated matching between predicted structures and experimental spectra, thereby improving the identification capability of trace-level unknowns [63,64]. Additionally, feature dimensions need to be expanded by incorporating cosmetic-relevant metrics such as “matrix tolerance” and “functional group-specific fragments”, while enabling dynamic weight adjustment of features based on product formulation characteristics. These enhancements will collectively improve the adaptability and accuracy of the algorithm across diverse types of cosmetic matrices.

## 6. Conclusions and Future Perspectives

MN technology has become an important tool for ensuring the safety of cosmetic products and conducting research and development of their efficacy, thanks to its efficiency in non-targeted screening and accuracy in structural annotation. For safety regulatory purposes, this technology will enable the creation of a closed-loop risk screening system spanning the entire supply chain from raw materials to finished products. In efficacy research and development, analyzing metabolic changes in individuals with different skin types after product use and combining them with molecular network mining for correlation analysis can provide a molecular-level basis for developing personalized skincare products.

Regarding technical breakthroughs, the focus will be on addressing two major challenges: complex matrix interference and annotation gaps. To reduce matrix interference caused by components such as surfactants and preservatives in cosmetics, detection sensitivity for trace prohibited ingredients will be enhanced through optimized pretreatment methods, adjusted MS ion source parameters, and improved separation capabilities using ion mobility mass spectrometry. Additionally, a cosmetics-specific spectral database containing prohibited substances and functional ingredients will be developed to fill annotation gaps.

At the level of integration and innovation, the combined use of AI and detection technologies will be improved. Machine learning and deep learning algorithms hold significant potential in MN-based cosmetic analysis. Specifically, deep learning algorithms will prove pivotal in analyzing spectra with low matching rates, effectively reducing omissions in unknown substance identification. Beyond this, AI will underpin end-to-end intelligence in MN workflows: machine learning can refine clustering rules, enable automated detection of suspicious substances, and generate real-time risk warnings, elevating analytical efficiency and accuracy. Looking ahead, the integration of MN with AI and machine learning will not only improve analytical accuracy but also strengthen compliance monitoring and support adaptive regulatory frameworks. This evolution will continue to propel the cosmetics industry toward a future defined by precision, intelligence, and consumer-centric innovation.

## Figures and Tables

**Table 1:** Comparative analysis of molecular networking technologies.

Technology	Core Principle	Advantages	Limitations	Ideal Applications
CLMN	Clusters compounds via direct comparison of MS^2^ spectral fragment ion similarity	Simple algorithm; rapid component grouping	Cannot resolve isomers; no quantitative capability; noise-sensitive (low-abundance ions)	Preliminary screening of mixtures
FBMN	Integrates RT, ion mobility, isotope patterns, and other features	Improved isomer resolution; enhanced reliability in complex matrices; semi-quantitative analysis	Dependent on LC-MS preprocessing software (e.g., MS-DIAL 4); large data volumes	Fine-scale analysis of complex systems (e.g., plant extracts)
IIMN	Correlates different adduct ions (e.g., [M+H]^+^/[M+Na]^+^) via chromatographic peak shape correlation	Resolves adduct splitting; enhances annotation propagation; detects ion–ligand complexes	Database-dependent; limited for novel modifications	Multi-adduct systems (e.g., metabolite profiling)
BMN	Maps bioactivity data (anti-inflammatory/antimicrobial) onto molecular networks to locate active clusters	Rapid identification of bioactive compounds; guides targeted isolation; links structure to function	Requires additional bioassays; fails with unclear mechanisms	Bioactive ingredient discovery (e.g., cosmetic actives)
BBMN	Integrates biosynthetic rules with MN for selective filtering of structural domains	High selectivity for novel scaffolds; simplifies complex datasets; provides visual guidance for new structures	Relies on biosynthetic rule libraries; may miss non-canonical metabolites	Novel scaffold discovery (e.g., microbial secondary metabolites)

**Table 2:** Applications of MN in the discovery of active ingredients for cosmetics.

No.	Study Subject	Identified Compounds	Cosmetic Efficacy	Role of MN in Screening/Identification	Ref.
1	Three Polynesian plants	Glycosylated flavonols, phenolic acids, C-flavonoids, iridoids, secoiridoids	Promoting dermal papilla cell proliferation (hair care)	BMN for identifying bioactive metabolites	[70]
2	Five Polynesian medicinal plants	Quercetin-*O*-rhamnoside, rosmarinic acid, curcumin (61 metabolites total)	Antioxidant (anti-photoaging)	LC-MS/MS with MN for identifying seven key phenolic radical scavengers	[71]
3	*Celastrus orbiculatus* fruits	12 novel dihydro-*β*-agarofuran sesquiterpenes; 15 known compounds	Melanin inhibition (whitening)	BMN for discovering bioactive ingredients	[72,73]
4	Marine red algae	Mycosporine-like amino acids	UV protection and antioxidant (sunscreen)	UHPLC-HRMS with FBMN and GNPS workflow enabling high-throughput dereplication	[74]
5	*Arthrospira platensis* and *Chlorella vulgaris*	Lys-Val, Val-Arg, Tyr-Phe, Leu-Gly-Leu (8 di/tri-peptides)	Antioxidant and anti-aging	MS-based GNPS networking identifying key bioactive peptides	[75]
6	Nine Apiaceae fruits	Apigenin and derivatives	Antioxidant and lipogenesis inhibition (anti-cellulite)	UPLC-HRMS with MN for activity screening	[76]
7	Three French oak extracts	Quercetin derivatives, ellagic acid, procyanidin B_2_, condensed tannins, flavonol glycosides	Collagenase inhibition and ROS scavenging (anti-aging)	UHPLC-HRMS with MN for activity polyphenol screening	[77]
8	*Inula japonica* leaf extract	Inujaponics A-C, caffeoylquinic acids, caffeoylglucuronic acids	MMP-1 inhibition and collagen synthesis (anti-aging)	LC-MS with MN for identifying caffeoylglucaric and caffeoylquinic acids	[78]

## Data Availability

No new data were created or analyzed in this study. Data sharing is not applicable.

## Abbreviations

The following abbreviations are used in this manuscript:

AI	Artificial intelligence
BBMN	Blocks-based molecular network
BMN	Bioactive molecular network
CAS	Chemical Abstracts Service
CLMN	Classic molecular networking
DPPH	2,2-Diphenyl-1-picrylhydrazyl
FBMN	Feature-based molecular networking
FRAP	Ferric reducing antioxidant power assay
GC-MS	Gas chromatography-mass spectrometry
GNPS	Global Natural Product Social Molecular Networking
HILIC	Hydrophilic interaction liquid chromatography
HPLC	High-performance liquid chromatography
HPLC-MS/MS	High-performance liquid chromatography–tandem mass spectrometry
HRESIMS	High-resolution electrospray ionization mass spectrometry
HRMS	High-resolution mass spectrometry
IIMN	Ion identity molecular networking
LC-MS/MS	Liquid chromatography–tandem mass spectrometry
LC-Q-TOF-MS	Liquid chromatography-quadrupole-time-of-flight-mass spectrometry
MeOH	Methanol
MN	Molecular networking
MSH	Melanocyte-stimulating hormone
MS/MS	Tandem mass spectrometry
MS1	First-stage mass spectrometry
MS2	Second-stage mass spectrometry
NIST	The National Institute of Standards and Technology
NMR	Nuclear magnetic resonance
ROS	Reactive oxygen species
QuEChERS	Quick, easy, cheap, effective, rugged and safe
TOF	Time of flight
